# A Case Report of Mycosis Fungoides Presenting With Blister Formation

**DOI:** 10.7759/cureus.54213

**Published:** 2024-02-14

**Authors:** Hirofumi Kawamoto, Natsuko Saito-Sasaki, Yumiko Sakuragi, Yu Sawada

**Affiliations:** 1 Dermatology, University of Occupational and Environmental Health, Kitakyushu, JPN

**Keywords:** prodromal symptom, histological analysis, case report, blister, mycosis fungoides

## Abstract

Mycosis fungoides (MF) is the most common primary cutaneous T-cell lymphoma with a usually indolent course. Early detection is crucial for effective intervention. We present a case of a 40-year-old male with MF exhibiting blistering as a rare precursor symptom. Despite initial treatment for eczema, the condition worsened over 10 months, leading to erythema, edema, and enlarged lymph nodes. Laboratory and imaging findings confirmed the diagnosis of MF. The patient responded partially to cyclophosphamide/doxorubicin/prednisone in combination with brentuximab vedotin (A-CHP) therapy. This case highlights the significance of recognizing blistering as a prodromal symptom for early detection and management of MF.

## Introduction

Mycosis fungoides (MF) is the predominant primary cutaneous T-cell lymphoma, and its typical clinical symptoms manifest as patch and plaque lesions that gradually progress into nodules and tumors [1,2]. Generally, MF exhibits an indolent clinical behavior; however, the advanced stages may prove intractable with current therapeutic options [3,4]. Therefore, early identification of the existence of MF is a crucial issue for clinicians to initiate treatment in the early stages of the disease. Herein, we present a case of MF that exhibited blistering before the manifestation of typical MF symptoms. This suggests that blistering may serve as an important precursor symptom, highlighting its potential significance for early detection by clinicians.

## Case presentation

**Figure 1:**
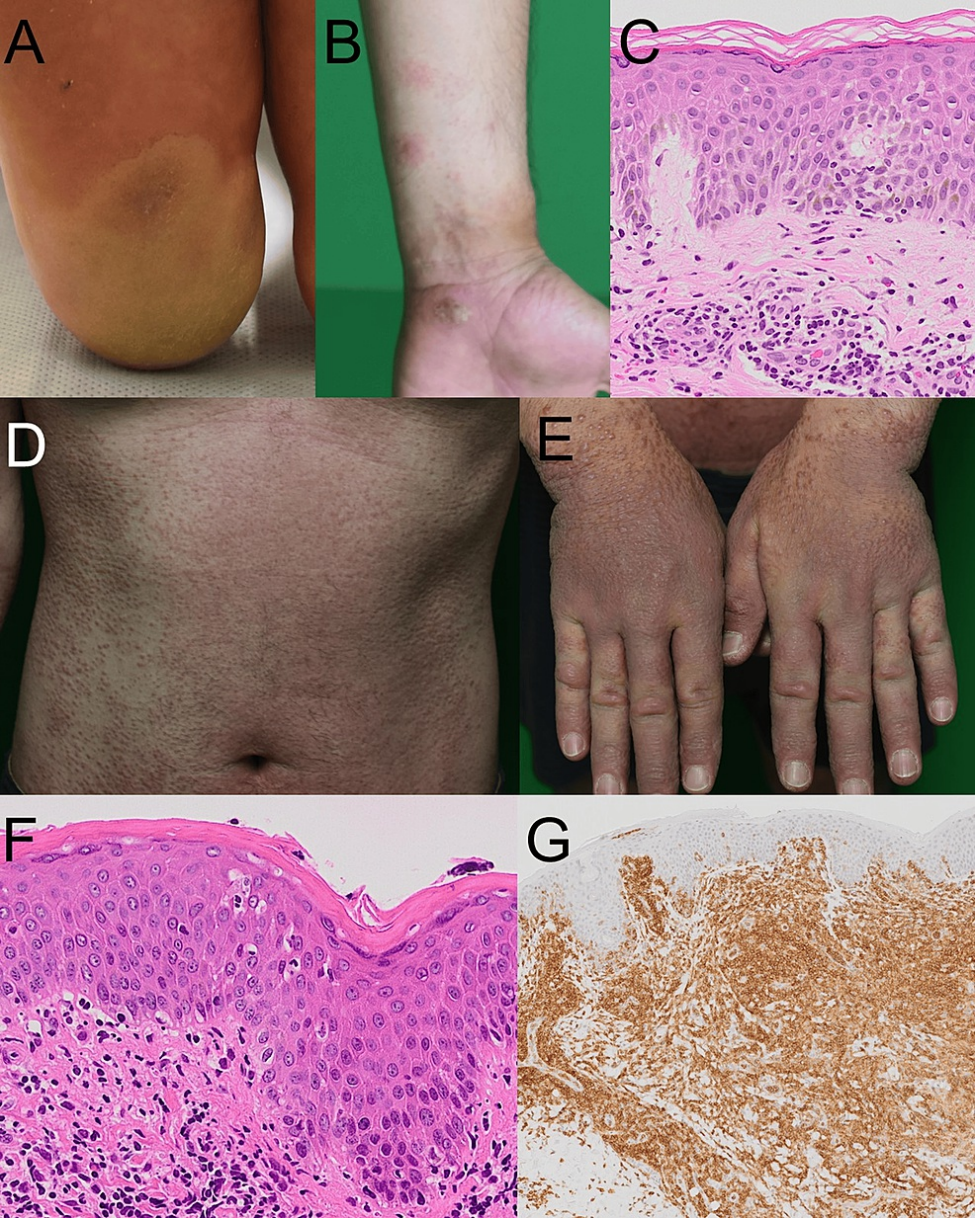
Clinical manifestation and histological examination A, B: Clinical manifestation of blister formation in heel (A) and arm (B). They exhibited tense blisters; C: Histological examination of the erythema before the onset of mycosis fungoides; D, E: Clinical manifestation of the onset of mycosis fungoides. Non-scaly infiltrated erythematous plaques were located on the trunk (D) and hands (E); F, G: Histological examination. Hematoxylin and eosin (H&E) staining (F). Atypical lymphocytes infiltrated into the epidermis and dermis. Immunostaining for CD4 (G). The atypical lymphocytes exhibited a CD4-dominant profile.

A 40-year-old male initially recognized recurring blisters on the right heel and erythematous lesions on the limbs. He was not on any medication. Despite undergoing initial treatment with topical steroids at a local dermatology clinic based on the diagnosis of eczema, the skin eruptions gradually worsened. He was referred to our department for the evaluation of his skin eruption two months after the initial presentation. The patient had no noteworthy medical history or prior medication use. A physical examination revealed that non-scaly infiltrated erythematous plaques were observed on the palms, forearms, soles, and lower legs (Figures 1A, 1B). The palms and soles exhibited tense blisters. A skin biopsy from the erythematous plaque revealed inflammatory cell infiltration, predominantly lymphocytes and eosinophils, in the dermis (Figure 1C). Direct immunofluorescence (DIF) examination revealed no deposition of immunoglobulins and complements. The patient was prescribed oral prednisolone 20 mg daily along with topical treatments as a possible diagnosis of eczematous eruption associated with auto-sensitized dermatitis at that time, initially resulting in the improvement of his skin eruption. Ten months later, the patient presented with erythema on the trunk and extremities, accompanied by significant edema on the arms and legs (Figures 1D, 1E). Enlarged elastic-hard lymph nodes were palpable in both inguinal areas. Laboratory findings indicated eosinophilic-predominant leukocytosis (9300/μl, eosinophil 13.2%), elevated lactate dehydrogenase (374 U/L), thymus‐and‐activation‐regulated chemokine (24800 pg/ml), and soluble IL-2 receptor (6375 U/ml). A skin biopsy showed clustering of small lymphocytes with irregular nuclei primarily in the upper dermis, infiltrating into the epidermis (Figure 1F). Immunostaining revealed positivity for CD3, CD4, and CD5, while CD8, CD20, and CD30 were negative (Figure 1G). Gene rearrangement unveiled T-cell receptor B-chain Cβ1 rearrangement, leading to the diagnosis of MF. A contrast-enhanced CT scan revealed enlarged lymph nodes in both axillae and inguinal areas. A biopsy of the right inguinal lymph node showed atypical lymphoid cells with bright cytoplasm and irregular nuclei. Following these examinations, the clinical stage was determined as T4NXM0B2. Subsequently, the patient underwent cyclophosphamide/doxorubicin/prednisone in combination with brentuximab vedotin (A-CHP) therapy at our hematology department, demonstrating efficacy with a partial response by the current treatment.

## Discussion

This case reports MF presenting with blisters, a rare manifestation initially reported in 1887 [5]. In all reported cases, blisters were observed concurrently with MF [5]. MF with blisters has a high one-year mortality rate, typically following a progressive course [5]. The mechanism of blister formation remains incompletely understood, with possibilities including the formation of subepidermal blisters due to the convergence of Pautrier microabscesses or the loss of adhesion between basal keratinocytes and the basal layer due to the proliferation of tumor T lymphocytes [6]. However, our case presents a unique aspect with the pre-existence of blisters preceding the typical manifestation of MF in this patient. MF typically manifests as patches, plaques, and tumors on the skin. In the early stages, it may resemble eczema or other skin conditions. Over time, the tumor progresses to more advanced forms, with infiltrated plaques and nodules. The clinical course is typically a slowly progressing chronic condition to more aggressive forms such as nodules or tumors with systemic involvement.

Upon the initial presentation, we entertained the differential diagnoses of bullous pemphigoid or allergic contact dermatitis. However, the sparse findings in the test results discouraged a conclusive indication of these conditions. This led us to contemplate the necessity of persisting with the treatment while keeping open the possibility of alternative diagnoses. The patient initially presented with blistering, showing intractable to topical steroids; however, later developed progressive erythema indicative of MF. Although several clinical manifestations, such as pityriasis lichenoides are recognized as possible prodromal symptoms [7], a crucial aspect in blistering conditions resistant to treatment lies in the prodromal symptoms, or they may be atypical for the initial manifestation, suggesting that MF had likely already developed in this patient.

## Conclusions

This case highlights the atypical presentation of MF with blistering as a rare precursor symptom, preceding the manifestation of typical MF symptoms. The patient initially presented with recurring blisters, resistant to topical steroids, which later progressed to erythema and lymphadenopathy. The unique aspect of blistering preceding MF underscores its potential significance as a prodromal symptom for early detection. Recognizing such atypical manifestations is crucial for clinicians, as early identification allows for timely intervention and improved outcomes in the management of MF. Further research is needed to elucidate the mechanisms underlying blister formation in MF and explore its implications for clinical practice.

## References

[1] Hwang ST, Janik JE, Jaffe ES, Wilson WH (2008). Mycosis fungoides and Sézary syndrome. Lancet.

[2] Jawed SI, Myskowski PL, Horwitz S, Moskowitz A, Querfeld C (2014). Primary cutaneous T-cell lymphoma (mycosis fungoides and Sézary syndrome): Part I. diagnosis: clinical and histopathologic features and new molecular and biologic markers. J Am Acad Dermatol.

[3] Olsen E, Vonderheid E, Pimpinelli N (2007). Revisions to the staging and classification of mycosis fungoides and Sezary syndrome: a proposal of the International Society for cutaneous lymphomas (ISCL) and the cutaneous lymphoma task force of the European Organization of Research and Treatment of Cancer (EORTC). Blood.

[4] Olsen EA, Whittaker S, Kim YH (2011). Clinical end points and response criteria in mycosis fungoides and Sézary syndrome: a consensus statement of the International Society for Cutaneous Lymphomas, the United States Cutaneous Lymphoma Consortium, and the Cutaneous Lymphoma Task Force of the European Organisation for Research and Treatment of Cancer. J Clin Oncol.

[5] Bowman PH, Hogan DJ, Sanusi ID (2001). Mycosis fungoides bullosa: report of a case and review of the literature. J Am Acad Dermatol.

[6] Kartsonis J, Brettschneider F, Weissmann A, Rosen L (1990). Mycosis fungoides bullosa. Am J Dermatopathol.

[7] Zaaroura H, Sahar D, Bick T, Bergman R (2018). Relationship between pityriasis lichenoides and mycosis fungoides: a clinicopathological, immunohistochemical, and molecular study. Am J Dermatopathol.

